# Supplementary figures and images for: Assessing the influence of culture on craft skills: A quantitative study with expert Nepalese potters
Source: PLoS One. 2020 Oct 1;15(10):e0239139. doi: 10.1371/journal.pone.0239139 (PMC7529208; doi:10.1371/journal.pone.0239139)

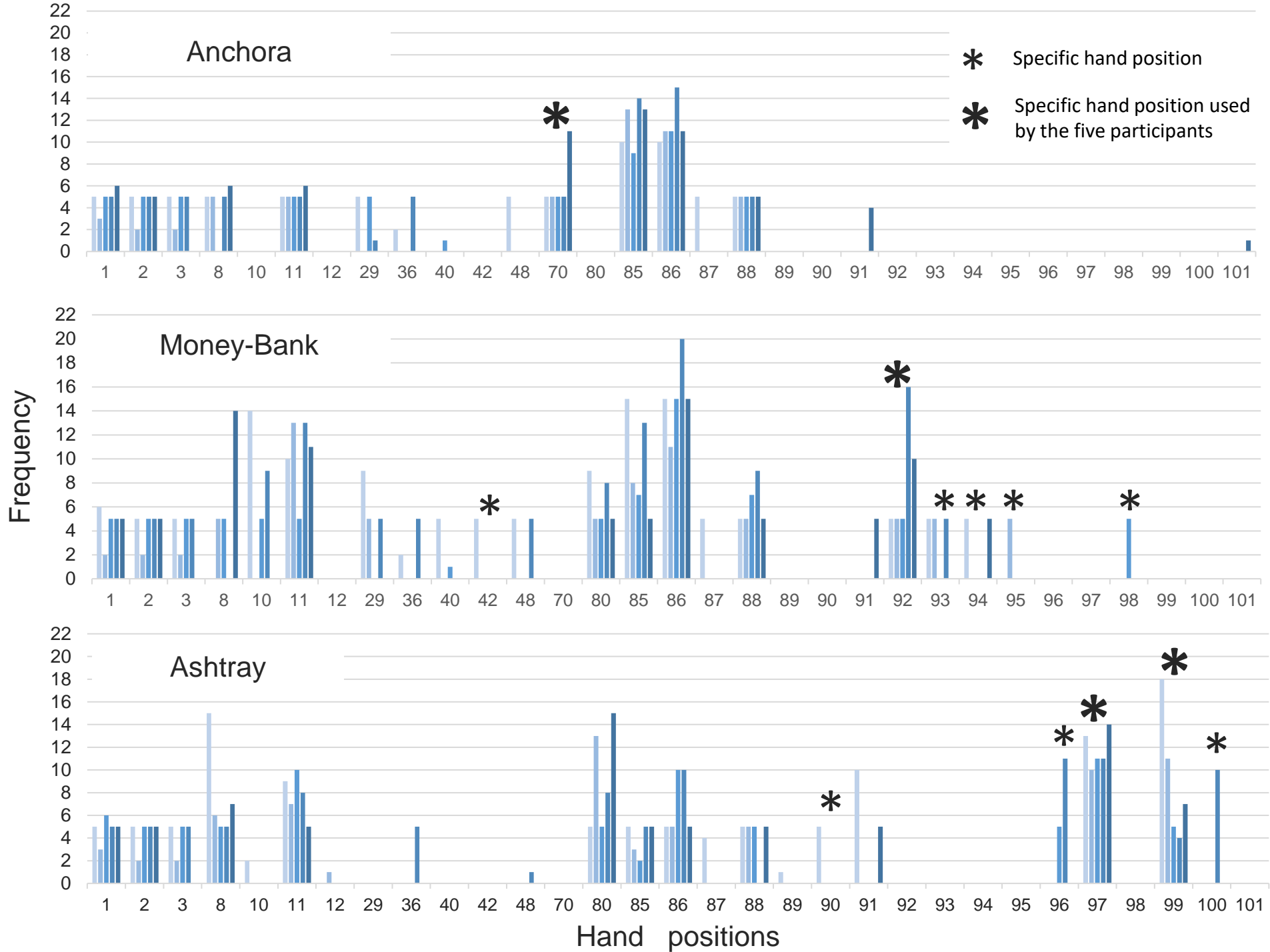

Supplement: S1 Fig — Bottom panel: Ashtray, middle panel: Money-Bank, top panel: Anchora. The graphs present the data of the five participants separately. On the x-axis each position is noted by its ethogram number. For each participant and each pottery type, the frequency of a given hand position corresponds to the total number of times this position was used throughout the five fashioning sessions (i.e. five trials). (PDF) [file pone.0239139.s001.pdf]

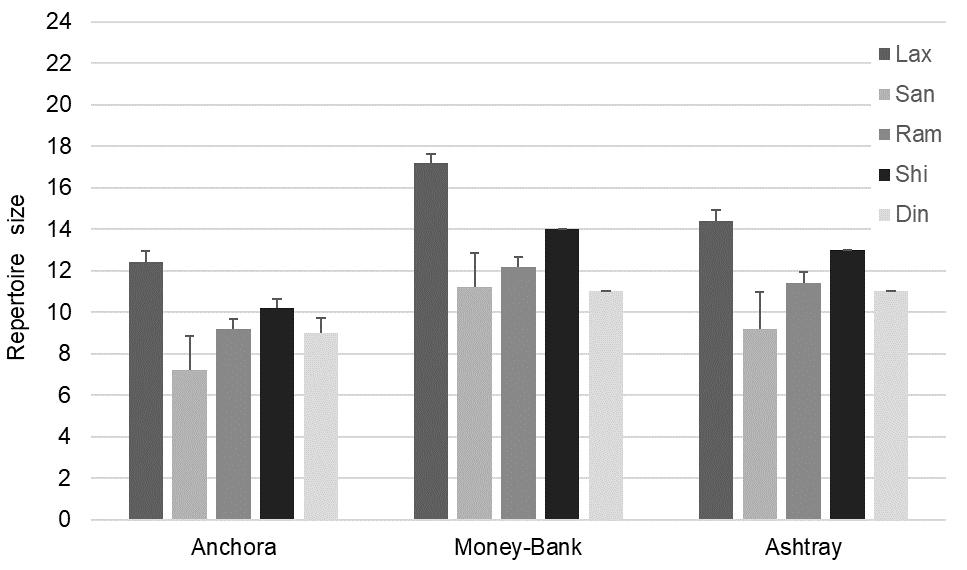

Supplement: S2 Fig — This graph shows the total number of different hand positions used by each potter for each pottery type separately. Error bars indicate the standard error from the five trials of each participant. (TIF) [file pone.0239139.s002.tif]
