# Supplementary material for: Assessing the influence of culture on craft skills: A quantitative study with expert Nepalese potters
Source: PLoS One. 2020 Oct 1;15(10):e0239139. doi: 10.1371/journal.pone.0239139 (PMC7529208; doi:10.1371/journal.pone.0239139)
Supplement: S3 Fig — The two first plots present the sequences of SAN and his father LAX producing an Anchora (first trial). The five following plots present the sequences of DIN (also son of LAX) producing the five Anchora (five trials). (PDF) [file pone.0239139.s003.pdf]

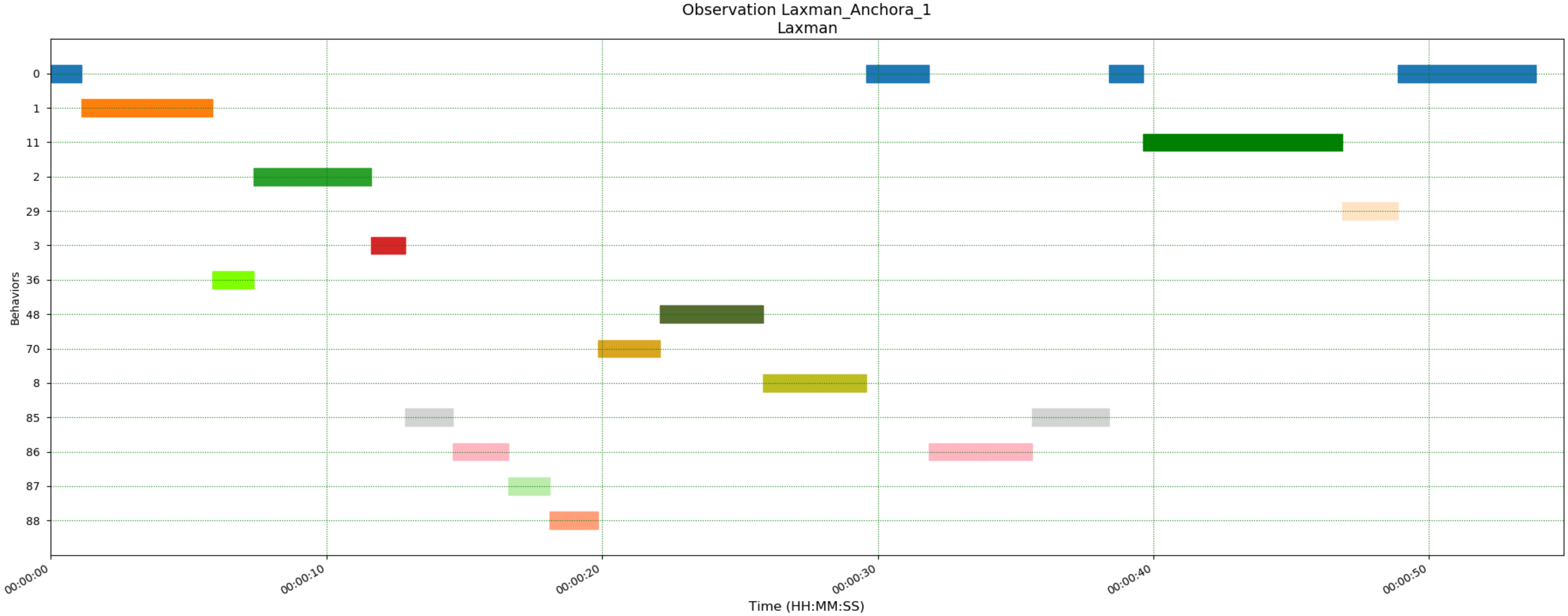
Observation Laxman_Anchora_1
Laxman
Behaviors
0
1
11
2
29
3
36
48
70
8
85
86
87
88
00:00:00
00:00:10
00:00:20
00:00:30
00:00:40
00:00:50
Time (HH:MM:SS)

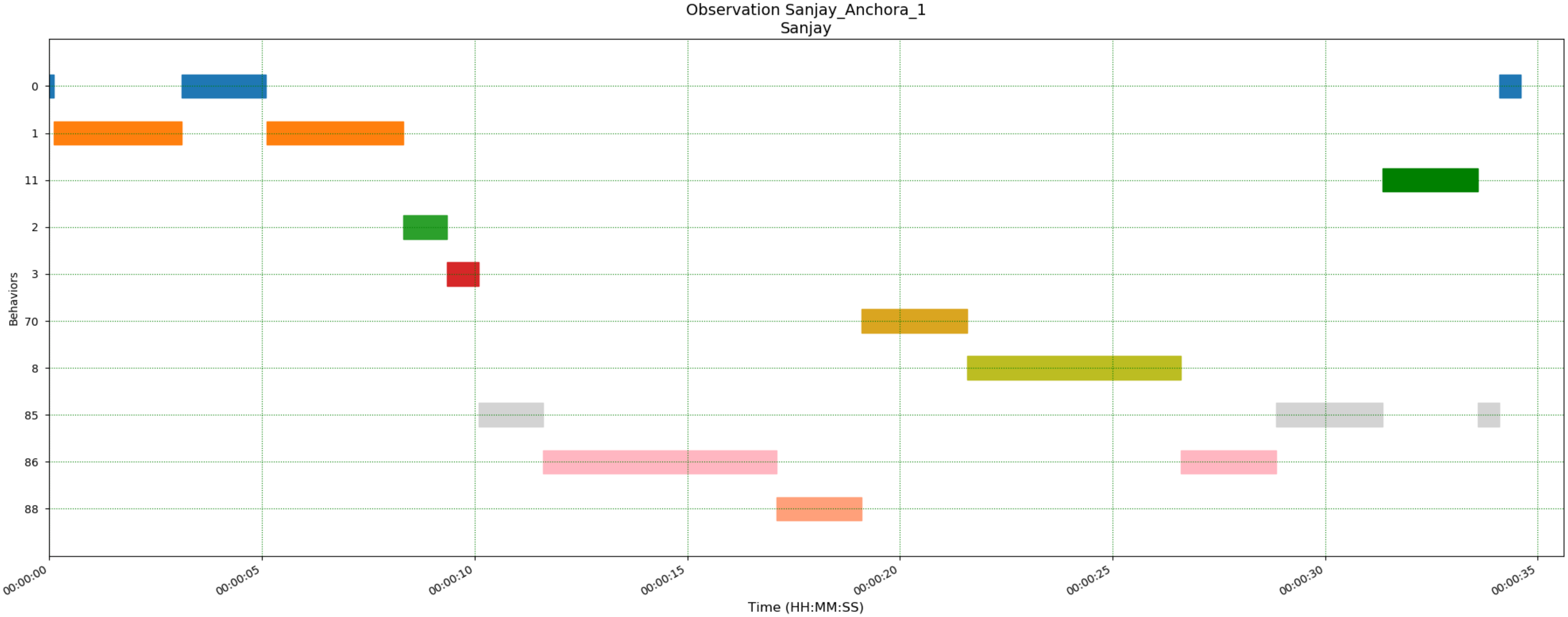
Observation Sanjay_Anchora_1
Sanjay
Behaviors
0
1
11
2
3
70
8
85
86
88
00:00:00
00:00:05
00:00:10
00:00:15
00:00:20
00:00:25
00:00:30
00:00:35
Time (HH:MM:SS)

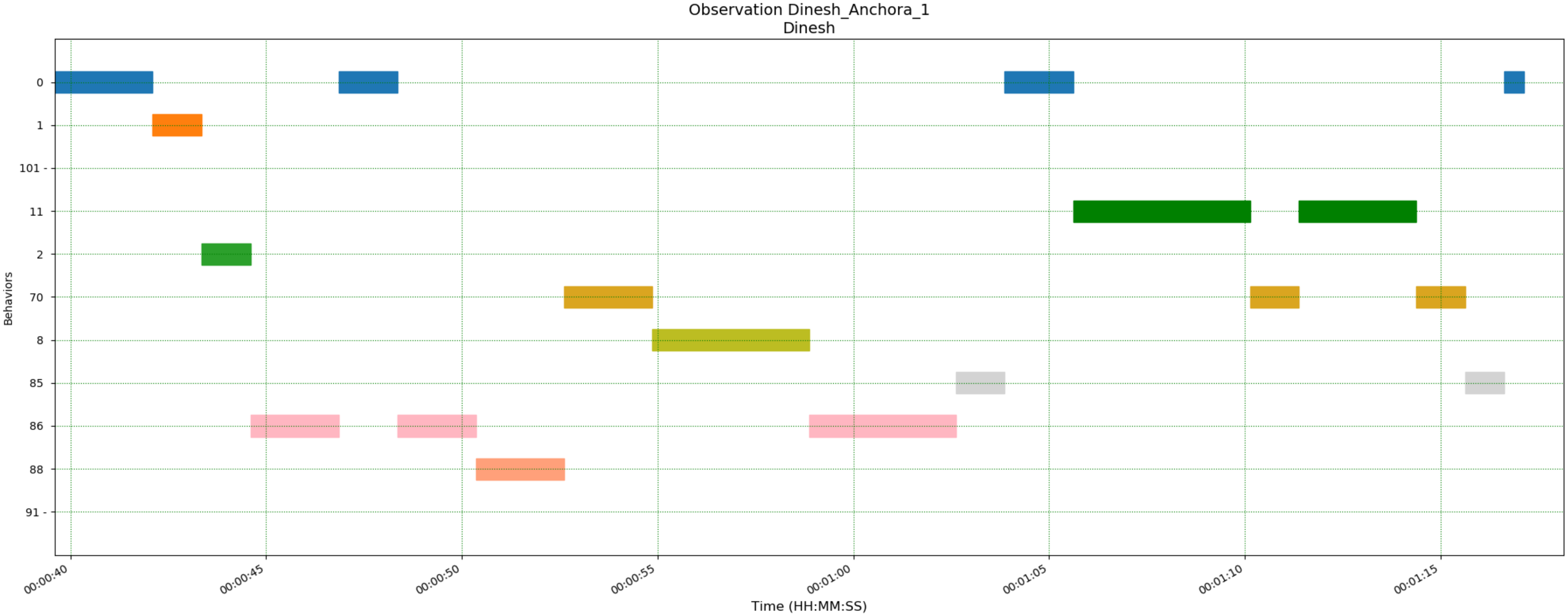
Observation Dinesh_Anchora_1
Dinesh
Behaviors
0
1
101
11
2
70
8
85
86
88
91
00:00:40
00:00:45
00:00:50
00:00:55
00:01:00
00:01:05
00:01:10
00:01:15
Time (HH:MM:SS)

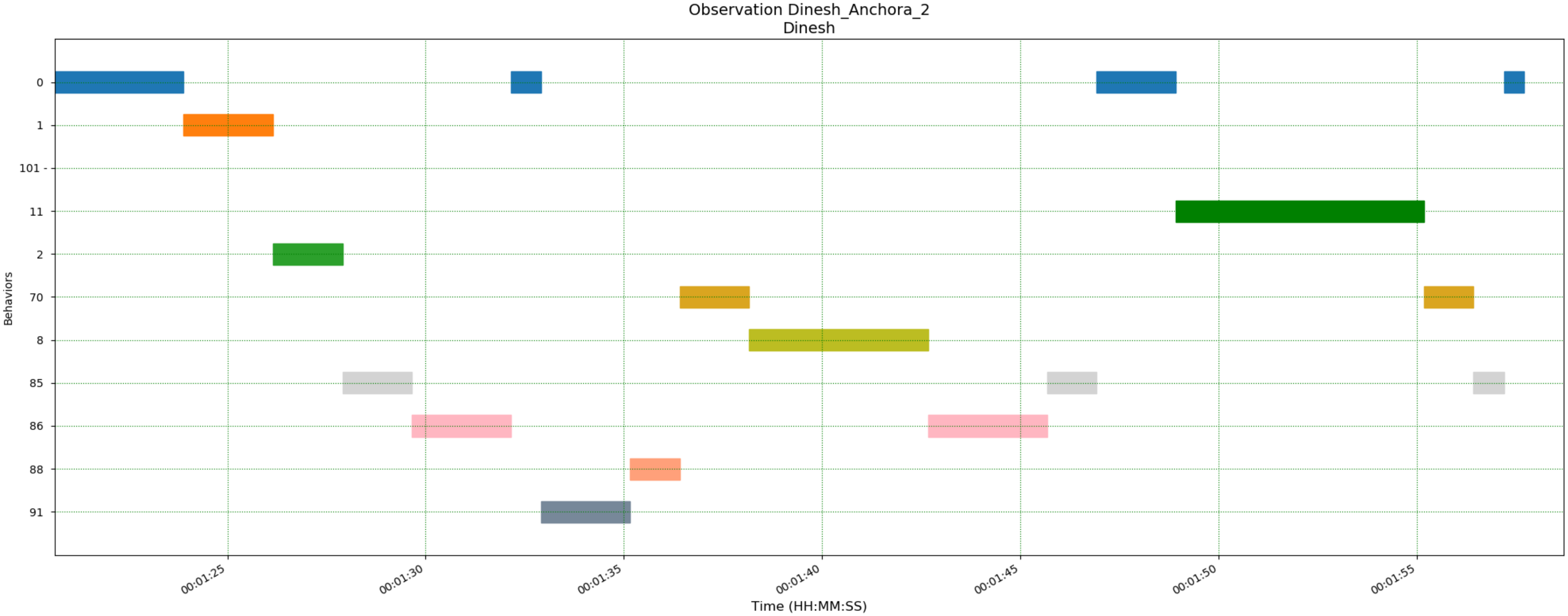

Observation Dinesh_Anchora_2
Dinesh
Behaviors
0
1
101
11
2
70
8
85
86
88
91
00:01:25
00:01:30
00:01:35
00:01:40
00:01:45
00:01:50
00:01:55
Time (HH:MM:SS)

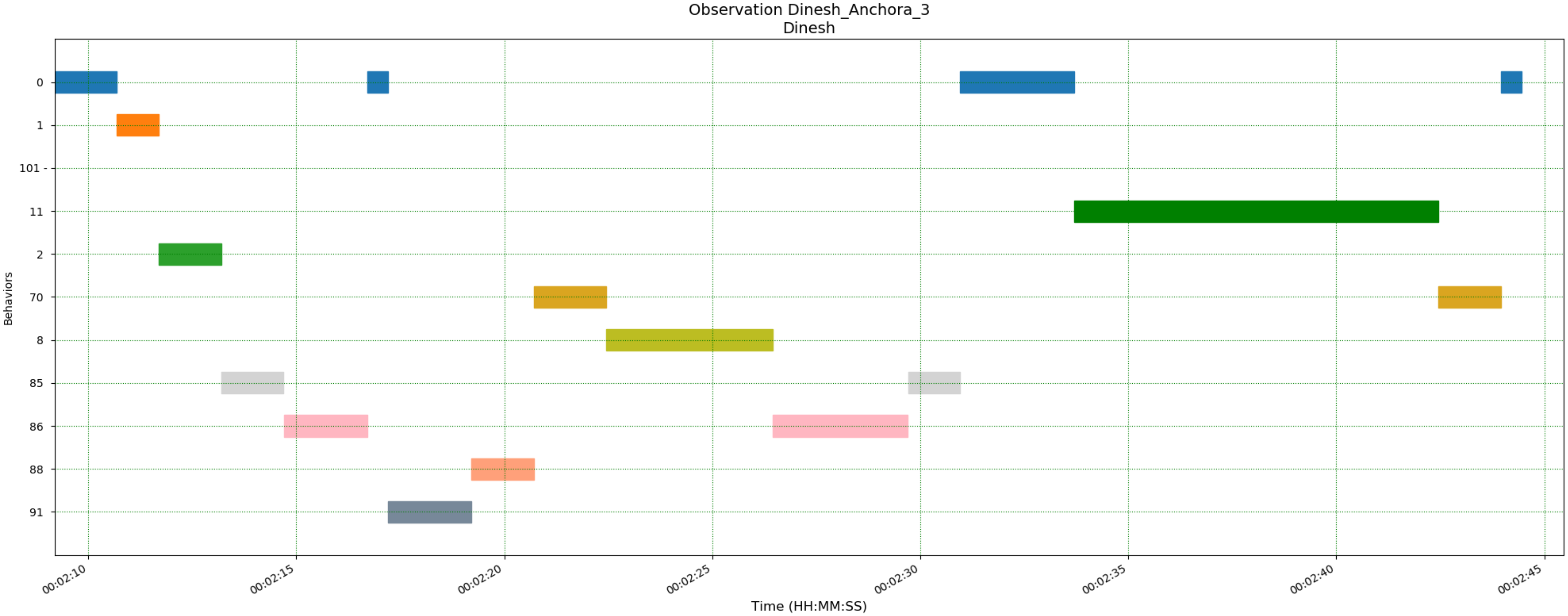
Observation Dinesh_Anchora_3
Dinesh
Behaviors
0
1
101
11
2
70
8
85
86
88
91
00:02:10
00:02:15
00:02:20
00:02:25
00:02:30
00:02:35
00:02:40
00:02:45
Time (HH:MM:SS)

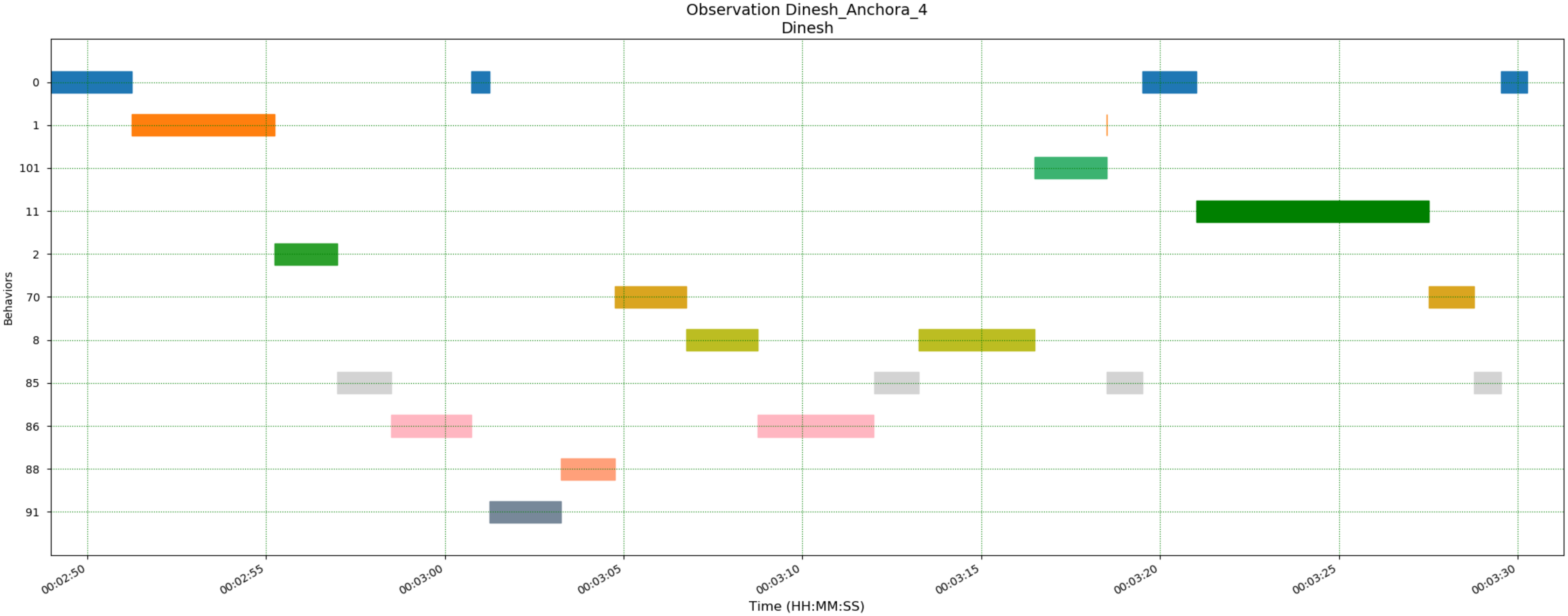

Observation Dinesh_Anchora_4
Dinesh
Behaviors
0
1
101
11
2
70
8
85
86
88
91
00:02:50
00:02:55
00:03:00
00:03:05
00:03:10
00:03:15
00:03:20
00:03:25
00:03:30
Time (HH:MM:SS)

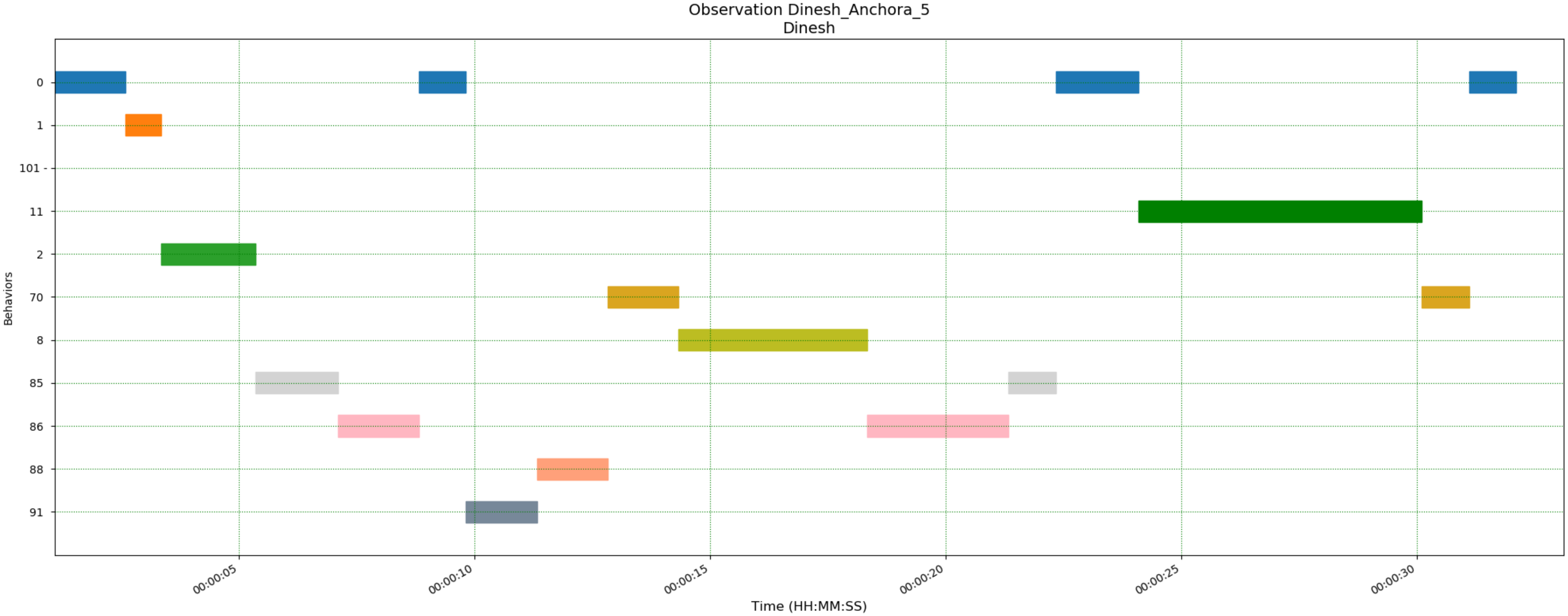

Observation Dinesh_Anchora_5
Dinesh
Behaviors
0
1
101
11
2
70
8
85
86
88
91
00:00:05
00:00:10
00:00:15
00:00:20
00:00:25
00:00:30
Time (HH:MM:SS)
