# Supplementary material for: Assessing the influence of culture on craft skills: A quantitative study with expert Nepalese potters
Source: PLoS One. 2020 Oct 1;15(10):e0239139. doi: 10.1371/journal.pone.0239139 (PMC7529208; doi:10.1371/journal.pone.0239139)
Supplement: S1 Table — We ran ANOVAs on the fashioning duration, number of fashioning gestures, and repertoire size (see the results in the manuscript). To identify significant differences between the three pottery types, we ran paired t-tests post-hoc with Tukey multiple testing correction. (DOCX) [file pone.0239139.s004.docx]

|  | Pair | *t* | *df* | *p* |
| --- | --- | --- | --- | --- |
| Fashioning duration | Anchora - Money-Bank | -5.11 | 8 | 0.002 |
|  | Anchora - Ashtray | -1.59 | 8 | 0.304 |
|  | Ashtray - Money-Bank | -3.52 | 8 | 0.019 |
| Number of gestures | Anchora - Money-Bank | -5.08 | 8 | 0.002 |
|  | Anchora - Ashtray | -3.25 | 8 | 0.028 |
|  | Ashtray - Money-Bank | -1.83 | 8 | 0.220 |
| Repertoire size | Anchora - Money-Bank | -8.72 | 8 | <0.001 |
|  | Anchora - Ashtray | -5.45 | 8 | 0.002 |
|  | Ashtray - Money-Bank | -3.27 | 8 | 0.027 |
