# Supplementary material for: Assessing the influence of culture on craft skills: A quantitative study with expert Nepalese potters
Source: PLoS One. 2020 Oct 1;15(10):e0239139. doi: 10.1371/journal.pone.0239139 (PMC7529208; doi:10.1371/journal.pone.0239139)
Supplement: S3 Table — This repertoire represents all the different positions used by a potter in the whole experiment. Between-potter mean and standard deviation are indicated in the two last columns. (DOCX) [file pone.0239139.s006.docx]

| Lax | San | Ram | Shi | Din | Mean | *sd* |
| --- | --- | --- | --- | --- | --- | --- |
| 25 | 17 | 18 | 20 | 15 | **19.00** | *3.81* |
