# Supplementary material for: Assessing the influence of culture on craft skills: A quantitative study with expert Nepalese potters
Source: PLoS One. 2020 Oct 1;15(10):e0239139. doi: 10.1371/journal.pone.0239139 (PMC7529208; doi:10.1371/journal.pone.0239139)
Supplement: Attachment — Submitted filename: Rebuttal-Letter.docx [file pone.0239139.s008.docx]

PloS ONE	London, July 21, 2020

Dear Editor,

We are grateful to the reviewers for their constructive comments. As detailed below, we have taken into account all the points raised. The manuscript has been modified to make clearer the message of our article. We believe these revisions will considerably improve the potential impact of our contribution.

**Reviewer #1:**

**1. Discussion of observed potting gestures between father and son (if son did indeed learn from father), or other instance where vertical transmission would imply the novice-expert relationship which is described for future research in the discussion section.**

We have now added in the Method section that the two brothers SAN and DIN learnt the skill from their father LAX. We also clarified in the Discussion that the skill of LAX was distinct to those of his two sons SAN and DIN. This demonstrates that the skill is not perfectly copied by the learner during the cultural transmission of the skill.

**2. Consideration of the ergonomic underpinnings of gestural variation. Some data collected relating to arm, hand, and finger dimensions may reveal potential grounds for deviation from learned gestures within novice-expert relationships. Could the ergonomic represent a further example of transcultural task constraint?**

**The authors describe potential for research into the long term gestural choices as a novice learns from an expert, and it is unclear whether any of the potters included in this study previously had this kind of relationship between them. If this is the case, the authors could provide some insight into the relationship between the gestures between potters who once had this novice-expert relationship in order to identify further meaningful gestural practices in their crafting behaviors. Presumably this can be included in the final manuscript without further need for fieldwork, and is recommended.**

**The consideration of ergonomic underpinnings for gestural variation is not addressed throughout the manuscript, but it would require further fieldwork (if not collected in initial fieldwork), a brief description of this variable is worth including.**

**Future work on this topic would do well to consider the way that an individual’s physical dimensions can facilitate or restrict the effectiveness of particular gestures which are learned during novice-expert relationships.**

In line with your suggestions, we have now described in the Discussion that physical ergonomics could influence the potters’ fashioning behaviours and their transmission during the learning process. As answered in the first comment, we have now detailed the tutor-novice relationship between LAX and his two sons and the fact that their hand positions (repertoire and sequences) were different.

**Reviewer #2:**

**1. Terminology: From my perspective several terms should be better clarified: the concept of ‘motor skills’ and what it entails is unclear, and many will be distracted on the overuse of the concept and its context. It is also worthy to explain what the authors mean by ‘fashioning operations’.**

We acknowledge that the concept of ‘motor skill’ is not widely shared in archaeology and may be confusing. This concept comes from the motor behaviour field where our study is grounded. We have now indicated in the Introduction that ‘motor skill’ is synonym of ‘motor habits’, a term which is more familiar in archaeology and anthropology.

We have removed the word ‘operation’ to clarify the sentence (line 62).

**2. Figures:**

**- Figure 1 is problematic. I don’t understand it. Why is task equated to goal? The arrows are leading to directions which are not clear. Not all the relations are explained especially their location in the flow diagram.**

We have detailed in the caption of Figure 1 why the task is considered as a ‘goal’ in the motor behaviour theoretical framework. The meanings of the different arrows have been made clear through additional sentences indicating the relations between the different parts of the diagram.

**- The experiment is conducted by expert Nepalese potters, why the hands/gestures from the Indian experiment are presented in Figure 2? I suggest that the authors will provide a new figure incorporating Nepalese gestures.**

We now provide a Figure 2 with Nepalese hand positions.

**- In the text supplementary figures are inserted. I believe that eventually they will be included in the supplementary material. I suggest that some of the figures will be listed in the main article – pulled back to the main text.**

All the supplementary figures and tables which are indicated in the text are now listed at the end of the article.

**- The absolute N values are missing in Figure 6. This is important since in the discussion these numbers are mentioned: “…through 10 specific cultural hand positions (Fig 6)”**

We have added the absolute N values in the legend of the Figure 6 and indicated them in the caption of Figure 6.

**- Finally, my most important comment related to Figure 7. This figure is an important presentation of the manuscript results. Yet, it is crowded with information. I suggest finding an alternative way to demonstrate/visualize these results – For example, in Gandon et al 2013 the cumulative percentage graph was harnessed. I’m sure there are many other ways to illustrate the distribution of gestures.**

We are aware that it is not easy to extract information from Figure 7 which contains a lot of information. What we intend to show with this figure is the detailed frequency of each hand position, for each potter, underlying the positions which were cultural and those which were individual (by the black and grey asterisk). The cumulative percentage graphs could appear easier to grasp but in fact they are also misleading given their resemblance with time series data. They give to the reader the impression of hand position sequences and not the simple frequencies. Therefore, we decided to keep the figure as it is, adding details in the caption to help understanding.

**3. Methodology - It is recommended to add additional information on the potters. In particular point to family relations, village proximity etc. Some of the sections in the methodology are not clear.**

We now clearly state in the “Participants and cultural setting” sub-heading that the participants involved in the experiment were all living and working in the pottery neighbourhood of Bhaktapur (Nepal, Kathmandu district). We also indicate the family relations between the participants.

**In particular, I recommend adding information on the Maxline procedure. In addition, it will be very helpful if this section will be divided to sub-topics (sub-headings).**

The section about the hand position sequences analysis has been re-written with additional information on the Maxline index (renamed *L_max_*). We have also added sub-headings in the Method section to make it correspond with the Results section.

**4. Results: As mentioned above, this study managed to provide very interesting results, as, “… even though wheel-throwing fashioning is highly constrained, there is still room for cultural and individual variations.” Or “similar the products are given the high degree of individual gestures. “or, “The developed skills are therefore likely to contain both cultural and individual traits (discussion, P.28). Yet isn’t this dependent on the cultural background. The variability between available cultural norms. Will we find the same individual signature in a culture with very strict cultural background? I suggest to tune this down. In general, the results should be more straightforward since in some sections the paper finds that craft skills lead to a cultural marking, yet it is also stated that the craft skills in not fully determined by a cultural marking. This point needs to be clarified and extended in this regard.**

We can assume, as suggested in this comment, that the individual and cultural influences on the manufacturing behaviour of craftsmen vary with the cultural context, notably because models of learning vary culturally (Rogoff, 1990, 1993). The more that learners are given responsibility and autonomy, the more diverse would be the fashioning patterns in a community of practice (with a significant part of individual hand positions). On the contrary, if tutors explicitly insist that their novices accurately reproduce their gestures, a given set of cultural hand positions can perpetuate in lineages of craftsmen. To test these hypotheses requires a concurrent analysis of the potters fashioning behaviours and the models of learning (through the teaching interactions between tutors and their novices). We have included this issue in the Conclusion to clarify the message of our article.

**5. The Archaeological viewpoint - It is not fully clear how this study can aid archaeologists in interpreting archaeological ceramic types. How is it possible to detect motor skills (that lead to cultural constraints) on the archaeological record? The authors state that it will be possible to identify traces throughout the vessel’s profiles (in case of fashioning techniques: wheel-throwing and wheel-coiling) – How? An archaeological example should be integrated to better clarify this point.**

We have revised the paragraph relating to the technological style to clarify the contribution of our study in the interpretation of archaeological vessels. We now refer to specific archaeological case studies.

**I recommend that the article will undergo final touch-ups in English editing**

As recommended, the article has been revised for English language.

We thank you in advance for the consideration given to this revised manuscript.

Sincerely,

Enora Gandon

(corresponding author)
